# Resting State Interhemispheric Motor Connectivity and White Matter Integrity Correlate with Motor Impairment in Chronic Stroke

**DOI:** 10.3389/fneur.2013.00178

**Published:** 2013-11-07

**Authors:** Joyce L. Chen, Gottfried Schlaug

**Affiliations:** ^1^Neuroimaging and Stroke Recovery Laboratories, Department of Neurology, Beth Israel Deaconess Medical Center, Harvard Medical School, Boston, MA, USA; ^2^Heart and Stroke Foundation Canadian Partnership in Stroke Recovery, Sunnybrook Research Institute, Toronto, ON, Canada; ^3^Department of Physical Therapy, University of Toronto, Toronto, ON, Canada

**Keywords:** corticospinal tract, transcallosal motor tract, motor recovery, resting state fMRI, DTI

## Abstract

Functional and structural reorganization in the brain occurs after stroke. The ability to predict motor outcomes may depend on patterns of brain functional and structural connectivity. We tested the hypothesis that alterations in motor transcallosal and corticospinal connections correlate with motor impairment in patients with chronic stroke. Eleven ischemic stroke patients underwent the Upper Extremity Fugl-Meyer (UE-FM) assessment, resting state functional magnetic resonance imaging, and diffusion tensor imaging (DTI). Twelve healthy control subjects underwent DTI. We assessed the temporal coupling in neural activity between interhemispheric motor cortex, and white matter integrity by means of fractional anisotropy (FA), in the transcallosal motor fibers and corticospinal tract. Partial correlation analyses were performed to determine whether these connectivity measures correlate with Upper UE-FM scores. Patients compared to controls had reduced FA in common voxels of transcallosal motor and ipsilesional corticospinal fibers. Within the patient group those with higher interhemispheric motor cortex connectivity and higher FA in the transcallosal motor fibers were less impaired. The results show that markers of functional and structural motor cortex connectivity correlate with motor impairment in the chronic stage of stroke.

## Introduction

Damage from ischemic stroke results in functional and structural reorganization of ipsilesional sensorimotor regions and their transcallosal as well as corticospinal connections, however, the role of transient or persistent contralesional changes is still a matter of active research (1–4). A highly germane question is whether these adaptations are related to a patient’s motor stroke outcome.

Resting state functional magnetic resonance imaging (fMRI) provides a measure of the functional organization of the brain and can been applied to study neural reorganization after stroke. Resting state connectivity between interhemispheric sensorimotor regions were found to correlate with scores on the Action Research Arm Test (ARAT), a measure of arm ability, in patients <4 weeks post-stroke (5). Other studies have shown that connectivity of ipsilesional M1 with various regions (e.g., thalamus, supplementary motor area (SMA), middle frontal gyrus, parietal lobule, and postcentral gyrus) positively correlate with increased scores on the Fugl-Meyer assessment of impairment, in individuals with acute and chronic stroke (6, 7). Experimental stroke models in rats whereby increases in functional gains were parallel by increases in interhemispheric sensorimotor connectivity (8) also support the human findings.

Studies have also explored the potential role of fractional anisotropy (FA) as a marker of white matter (WM) structural integrity in motor stroke recovery. Chronic stroke patients with higher levels of motor skill, as measured by the Purdue Pegboard test and maximum index finger tapping rate, have higher FA in the ipsilesional and contralesional CST (3). In addition, ARAT scores were found to positively correlate with FA in the transcallosal tracts connecting sensorimotor regions (4). Together these findings suggest the potential role of sensorimotor interhemispheric connectivity, and transcallosal and CST WM integrity as functional and structural markers of motor stroke recovery.

The main aim of the present study was to study whether resting state interhemispheric motor connectivity and WM integrity in the transcallosal motor fibers or corticospinal pathways correlate with motor impairment in chronic stroke patients as measured by the Upper Extremity Fugl-Meyer (UE-FM) score. In particular, the Fugl-Meyer assessment is a highly valid and reliable measure of impairment (9) that has been shown to be a very good predictor of upper extremity motor recovery (10). We chose this measure as opposed to an assessment of ability and/or function because it more likely reflects true motor recovery as opposed to compensation (11), and is a test battery that can be used on all patients independent of their impairment severity. Based on prior findings, we hypothesized that patients with higher measures of connectivity and WM integrity would be less impaired.

## Materials and Methods

### Participants

**Figure 1:**
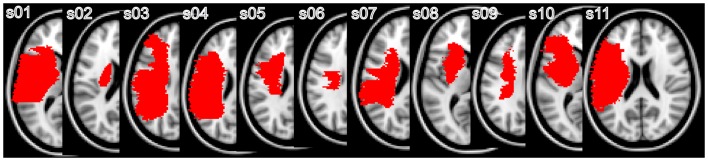
**Representative individual lesion maps: individual lesion maps are superimposed on the MNI standard template**. The slice closest to the internal capsule level with the greatest lesion is shown.

Eleven subjects who had a stroke (3 female) and 12 healthy control subjects (6 female) gave written informed consent to participate in the study that was approved by the Institutional Review Board (Table 1; Figure 1). Inclusion criteria for the stroke subjects were as follows: first ischemic stroke in the middle cerebral artery territory, no previous or subsequent cerebral ischemia or hemorrhage, no history of seizures, and presence of movement in the wrist (at least 10 degrees of dorsiflexion) and/or fingers (at least 10 degrees of flexion and extension). All patients underwent the UE-FM assessment that consists of 30 voluntary UE motions observed by a rater and 3 tendon tap responses, with a maximal score of 66. Healthy subjects had no history of neurologic or psychiatric disorders and were recruited to compare diffusion tensor imaging (DTI) derived measures between patients and healthy controls.

**Table 1:** **Patient characteristics**.

Patient	Age at assessment (years)	Sex	Hemisphere stroke	Time since stroke to assessment (months)	Lesion size (cm^3^)	UE-FM score
s01	45	M	L	15	173.8	10
s02	77	M	L	4	9.91	19
s03	54	F	L	11	189.1	17
s04	59	M	L	15	259.0	66
s05	63	M	R	26	96.1	20
s06	49	F	R	16	10.0	41
s07	51	M	L	9	92.9	66
s08	50	F	R	9	32.8	30
s09	64	M	L	20	32.6	15
s10	58	M	L	14	94.4	20
s11	63	M	L	15	154.7	66

Demographics for patients, identified as subject numbers (i.e., s01). M, male; F, female; L, left; R, right; UE-FM, Upper Extremity Fugl-Meyer score (maximum score = 66).

### MRI scan

Images were acquired on a 3-T GE scanner. All patients were scanned using resting state fMRI. T2^∗^ weighted EPI slices were acquired every 2 s (*TR* = 2 s, *TE* = 25 ms) for a total of 300 volumes. We obtained 28 horizontal slices covering the whole brain with an in-plane resolution of 3.75 mm × 3.75 mm, slice thickness of 4 mm, 1 mm gap (matrix size = 64 mm × 64 mm, field of view = 240 mm × 240 mm). Patients fixed their eyes on a red cross while a pulse oximeter recorded heart rate, and a pneumatic belt recorded respiration.

All patients and healthy control participants underwent DTI. Images were acquired with the following parameters: matrix size = 94 mm × 94 mm, field of view = 240 mm × 240 mm, 2.6 mm slice thickness, 56 slices. We acquired one set of 30 diffusion weighted images with a *b*-value = 1000 s/mm^2^, and 5 no diffusion weighted images.

High-resolution T1-weighted structural images were also acquired for all participants (resolution = 0.9375 mm × 0.9375 mm × 1.5 mm, matrix size = 256 mm × 256 mm^2^, field of view = 240 mm × 240 mm, *TE* = 2.8 ms, *TR* = 6.6 ms, flip angle = 15°).

### Analysis

All data were analyzed using FSL tools^1^ (12). Prior to any analyses, images for three stroke subjects who had right hemisphere lesions were flipped along the *x*-plane so that all lesions were now displayed on the left hemisphere.

#### Lesion

Lesions were drawn in each subject’s structural space on the T1-weighted images using the co-registered T2-weighted diffusion images as a reference for lesion extension. The lesion mask was non-linearly registered into standard space using FMRIB’s Non-linear Registration Tool (FNIRT) with an affine transformation (12 degrees of freedom). The normalized lesion mask was used to determine lesion volume. The lesion mask was also used during the registration steps of the structural, resting state fMRI, and DTI data so that voxels in the lesioned area would be excluded from the normalization procedure.

#### Resting state

##### Preprocessing

All images were first preprocessed using FMRIB’s Expert Analysis Tool (FEAT) software. The first six volumes were discarded to account for T1-saturation effects and to achieve steady state of the spin system. The images were then motion corrected by realignment to the middle volume of each run (13), spatially smoothed with a Gaussian kernel of 8 mm full-width at half maximum, high-pass temporal filtered at 0.01 Hz, and grand mean intensity normalized. Brain Extraction Tool (BET) was used to remove signal from non-brain tissue (14).

##### Nuisance variables

The time series for nuisance variables were computed and used as regressors of no interest. These included cerebrospinal fluid (CSF), WM, head motion, and physiological noise related to the cardiac cycle and respiration. To extract the time series for CSF and WM, we used FMRIB’s automated segmentation tool (FAST) to segment each individual’s high-resolution structural image. The resulting CSF and WM images were then eroded twice (i.e., two times the default eroding kernel, 3 × 3 × 3) using *fslmaths* to ensure that only voxels in the CSF and WM were included. These masks were then transformed into functional space using FLIRT (with 6 degrees of freedom) and their time courses extracted from the preprocessed data derived from the step above. The mean time course was calculated by averaging the time courses from all voxels within the mask.

Six motion parameters (*x*, *y*, and *z* translations and rotations) derived from motion correction during the preprocessing step were also obtained for each individual. Lastly, the PhLEM Toolbox (15)^2^ was used to generate regressors for the physiological data. We modeled the respiration and cardiac cycles, respiration volume and heart rate, using the *Retroicor* and *Variation* models in PhLEM.

##### Seed masks

Region of interest (ROI) masks in left and right primary motor cortex (M1) were derived using a combination of the precentral gyrus mask from the Harvard-Oxford atlas and the Jülich BA4a and BA4p masks (16). The Harvard-Oxford precentral masks were first thresholded by 20%, and binned. These masks also included the SMA. However, voxels inferior to *z* = 62 were clearly segregated to either the precentral gyrus or SMA. Due to the proximity of the precentral gyrus and SMA for voxels superior to *z* = 62, the SMA was defined as encompassing five voxels in the ±*x* direction from the midline; those voxels greater than ±5 voxels from the midline were considered to be part of the precentral gyrus. The masks were overlaid on the MNI152 2 mm standard brain and voxels that were posterior to the central sulcus were also excluded. The Jülich BA4a and BA4p masks were thresholded at 1% and the anterior boundary then used to segregate the precentral gyrus into primary motor cortex and premotor cortex (remaining portion of precentral gyrus). These masks in standard space were then non-linearly transformed into the functional space of each subject, using FNIRT. We also defined ROIs in bilateral parahippocampal gyrus from the Harvard-Oxford atlas to use as control regions. The prediction was that temporal coupling in neural activity between the left and right parahippocampal gyrus would not correlate with the UE-FM score. The posterior division of this region was modified by thresholding the masks by 20% and then binning them.

##### Resting state analysis

In a second FEAT analysis, the raw images were processed as described in the preprocessing step above with the nuisance regressors also included in the model. Thus, the residuals from this analysis have all nuisance regressors removed. The mean time course of the left and right M1 was extracted from the residuals and a Pearson’s correlation was performed on this data. Thus for each subject, we obtained an *r*-value that represented the correlation in neural activity between left and right M1.

Partial correlation was then used to determine whether a participant’s resting state connectivity between left and right M1 correlated with their UE-FM score, while controlling for age, time since stroke, and lesion volume. The same procedure was also performed for the left and right parahippocampal gyrus. All statistics were performed using SPSS (version 17.0.2).

#### Diffusion tensor imaging

The diffusion data were preprocessed using FMRIB’s Diffusion Toolbox (FDT). We first performed a whole brain analysis of WM integrity between patients and controls, investigating FA, radial, and axial diffusivity measures. FA quantifies the degree to which water diffusion is restricted and is calculated based on the three eigenvalues of the diffusion tensor, L1, L2, and L3. Radial diffusivity is quantified by the average of the second and third eigenvalues and is a measure of diffusion in the perpendicular direction to the tensor. Axial diffusivity is quantified by the first eigenvalue and measures diffusion in the direction parallel to the long axis of the tensor. The data were corrected for head motion and eddy current distortion, and brain extracted using BET. FA, L1, L2, and L3 images were then created by fitting the data to a tensor model at each voxel using DTIFIT within the FDT toolbox. We masked out the lesion in each subject’s data so that all analyses are performed on non-lesioned data.

Tract-based spatial statistics (TBSS) was next used to perform whole brain voxel-wise statistical analysis on the FA data (17) in patients and controls. All FA data were non-linearly registered into standard space using FNIRT. The lesion mask was also applied to the patient data during this step to ensure good registration. Next, the mean FA image was created and thinned to create a mean FA skeleton that represents the center of all tracts common to the group. The mean FA skeleton was thresholded at 0.3 and each subjects’ aligned FA data was then projected onto it. Since we masked out the lesion in the patient data, we reduce the chance of spurious projections of FA; prior studies had not done this (3, 4). The same procedure was applied for the analysis of radial and axial diffusivity measures.

Statistical analyses were performed using non-parametric permutation testing (Randomize in FSL) with 5000 Monte Carlo simulations. We looked for differences in FA, radial, and axial diffusivity between patients and controls, accounting for age and lesion hemisphere as regressors of no interest in the GLM. The statistical threshold was set to *t* > 3.1, *p* < 0.05, family wise error (FWE) corrected, based on cluster thresholding.

The TBSS results yielded significant differences in measures of WM integrity between patients and controls in regions of interest in the ipsilesional CST and the transcallosal tract connecting left to right M1 (transcallosal M1–M1). FA values were extracted in these regions of interest and used to correlate with UE-FM scores. For the CST, we extracted FA values from the significant cluster found in the TBSS analysis. However, we were unable to do this for the transcallosal M1–M1 tract since this cluster encompassed other regions of the corpus callosum that connect regions other than M1–M1. Therefore, tractography using the FDT toolbox was performed to derive a canonical transcallosal M1–M1 tract from healthy control subjects. Masks were drawn in standard space on the FMRIB58 FA template. Left and right M1 masks were drawn on a single horizontal slice at the level of *z* = 55. A waypoint mask of the corpus callosum was drawn on a single sagittal slice at *x* = 0. An exclusion mask was drawn on a horizontal slice at *z* = 10 that encompassed the entire brain. Two tractography analyses were performed per healthy control subject; streamlines were seeded from left M1 and passed through the corpus callosum and ended at right M1, and vice versa. The resulting tracts were thresholded with a minimum value of 5 to exclude noise, added together, and binned. Across the healthy control subjects, all combined M1–M1 tracts were added, thresholded with a minimum value of 1, and binned. This group transcallosal M1–M1 canonical tract was then intersected with the mean FA skeleton mask derived from the TBSS analysis. Thus, this new mask encompasses the centers of the transcallosal M1–M1 tracts common to all participants. FA values were then extracted from this mask for the patients.

If the relationship between FA in these regions and UE-FM was significant, radial and axial diffusivity measures were also extracted and correlated with UE-FM scores to further understand the nature of the FA alterations. Lastly, we assessed the relationship between FA in the transcallosal M1–M1 tract and interhemispheric motor resting state connectivity. For all analyses, we used partial correlations to factor out age, time since stroke, and lesion volume. All statistics were performed in SPSS (version 17.0.2).

The JHU WM atlas as part of FSL was used to localize changes in WM integrity; if no region was identified with the atlas, we noted where regions of WM changes occurred relative to gray matter areas.

## Results

The mean age of patients (57.54 ± 9.08 years) was not significantly different from the mean age of controls (59.06 ± 9.15 years) (unpaired *t*-test: *t* = 0.399, *df* = 21, *p* = 0.694).

### Resting state

**Figure 2:**
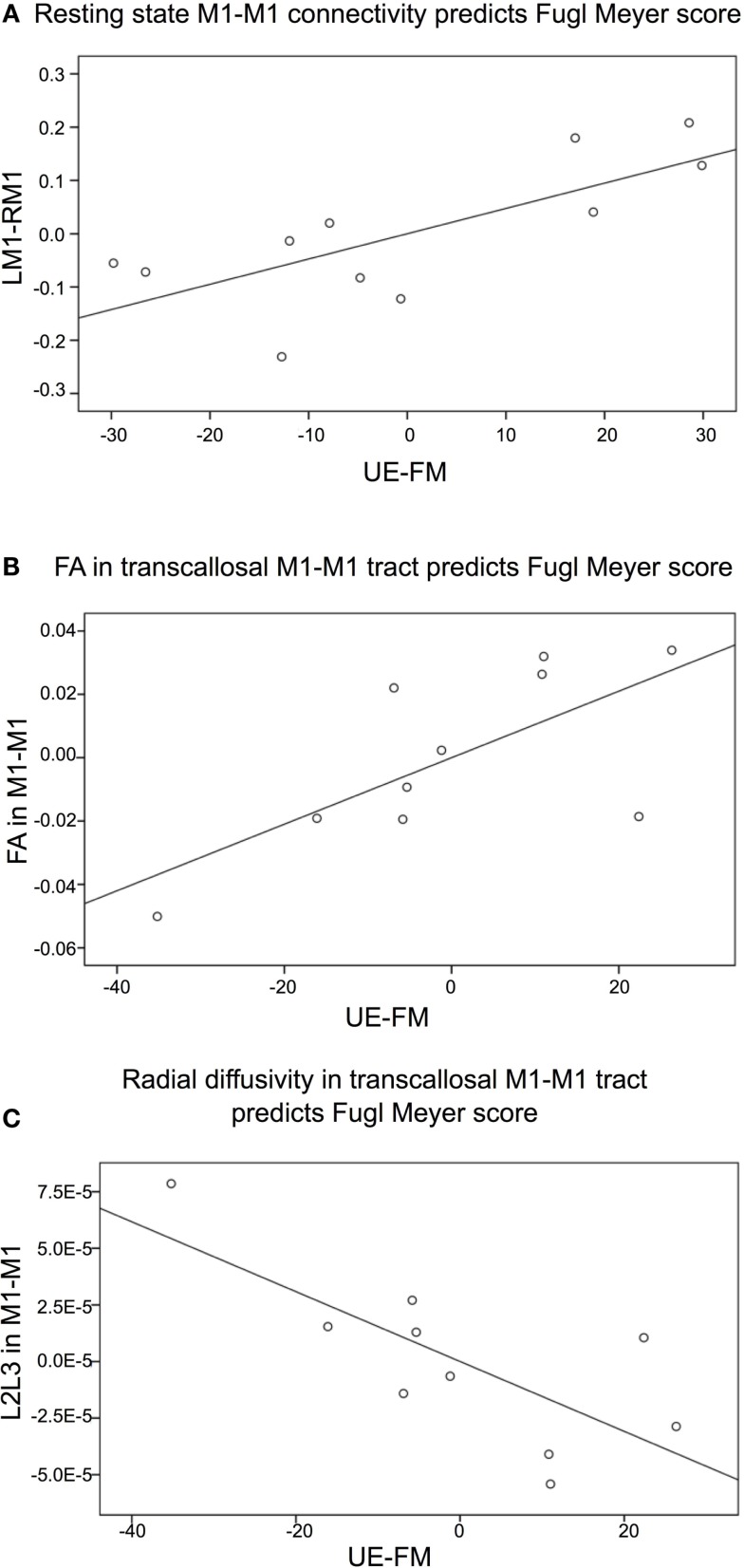
**Partial correlation graphs (plotted with standardized residuals) shows significant relationships between (A) resting state connectivity between left (L) and right (R) primary motor cortex (M1) and the Upper Extremity Fugl-Meyer (UE-FM) score, *R*^2^ = 0.55; (B) fractional anisotropy (FA) in the transcallosal tract connecting left and right primary motor cortex (M1–M1) and the UE-FM score, *R*^2^ = 0.48; (C) radial diffusivity (L2L3) in the transcallosal M1–M1 tract and the UE-FM score,*R*^2^ = 0.55**.

Partial correlation analysis showed that there was a significant positive relationship between resting state connectivity between left and right M1 and the UE-FM score, after controlling for age, time since stroke, and lesion volume (*r* = 0.741, *p* = 0.018 one-tailed, *df* = 6, *t* = 2.706) (Figure 2A). This indicates that participant’s with lower resting state connectivity between left and right M1 have lower UE-FM scores (i.e., those with more impairment). In contrast, there was no significant relationship between resting state connectivity between left and right parahippocampal gyrus and the UE-FM (*r* = −0.275, *p* = 0.255 one-tailed).

### Diffusion tensor imaging

**Figure 3:**
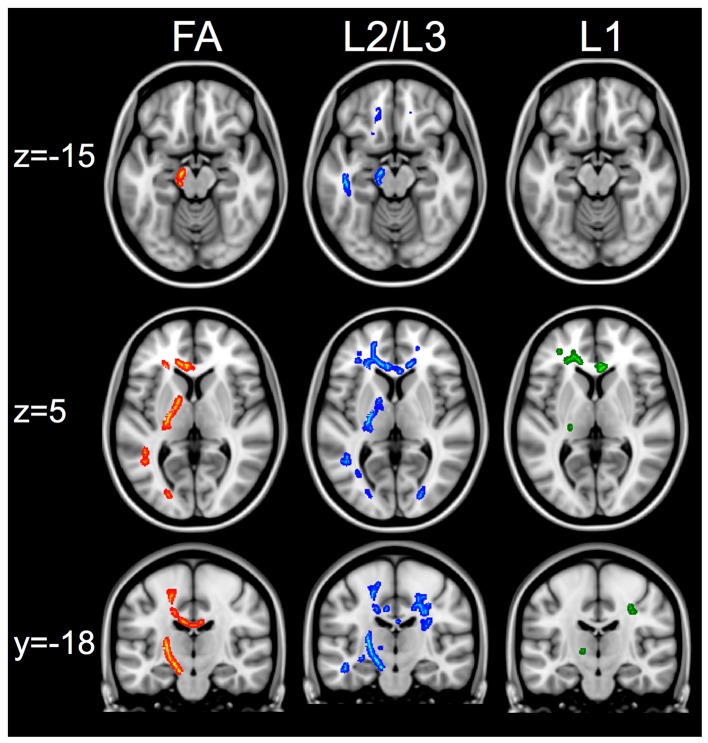
**White matter changes between healthy controls and patients**. First column shows regions of interest in the corticospinal and transcallosal M1–M1 tracts that have greater FA in controls compared to patients. Second column shows the same regions of interest that show greater radial diffusivity (L2/L3) in patients compared to controls. The third column shows that in the same regions, there are no changes in axial diffusivity (L1) measures when comparing patients versus controls. The skeletonized results are thickened to aid visualization of results. All images show results that are significant at *t* > 3.1, *p* < 0.05 FWE using cluster thresholding.

Patients had significantly lower FA values compared to healthy control subjects in several WM tracts not directly affected by the lesion. These alterations were located in the regions of interest identified *a priori*: the ipsilesional (L) CST (inferior to the lesion), encompassing the ipsilesional (L) posterior limb of the internal capsule (PLIC) and the ipsilesional cerebral peduncle, and the transcallosal M1–M1 tract (Figure 3). See Figure S1 and Table S1 in Supplementary Material for other regions of significant FA differences. There were no regions where patients had higher FA than control subjects.

Within the regions of interest identified *a priori*, patients also had significantly higher radial diffusivity (L2/L3) compared to controls and no differences in axial diffusivity (Figure 3). Patients also had significantly higher axial diffusivity in several areas outside the regions of interest (See Figure S1 and Tables S2 and S3 in Supplementary Material for lists of regions). There were no regions that had significantly higher radial or axial diffusivity in controls compared to patients.

Fractional anisotropy values from the patients were extracted from the transcallosal M1–M1 tract and correlated with UE-FM scores, controlling for age, time since stroke, and lesion volume. One patient was excluded from this analysis since this person had a high FA value albeit the lowest UE-FM score and the second lowest resting state M1–M1 connectivity value. This patient was also the only person who presented with flaccid tone of the UE; all remaining patients (*n* = 10) had some degree of spasticity. FA values in the transcallosal M1–M1 tract was significantly correlated with UE-FM scores; patients with lower FA values had lower UE-FM scores (*r* = 0.690, *p* = 0.043 one-tailed, *df* = 5, *t* = 2.132) (Figure 2B). With the outlier included the correlation was not significant (*r* = 0.328, *p* = 0.214 one-tailed). In conjunction, radial diffusivity values in the transcallosal M1–M1 tract was also significantly correlated with UE-FM scores; patients with higher radial diffusivity values had lower UE-FM scores (*r* = −0.744, *p* = 0.028 one-tailed, *df* = 5, *t* = −2.488) (Figure 2C). With the outlier included, the correlation was not significant (*r* = −0.407, *p* = 0.159).

Using partial correlation, FA values in the ipsilesional CST, distal to the lesion was not significantly correlated with the UE-FM score (with all patients *r* = 0.377, *p* = 0.179 one-tailed; with patient from above excluded *r* = 0.461, *p* = 0.149 one-tailed).

Fractional anisotropy in the transcallosal M1–M1 tract was not significantly correlated to resting state connectivity between left and right M1, after controlling for the effect of age, time since stroke, and lesion volume (*r* = 0.470, *p* = 0.144, one-tailed). The same patient was excluded from this analysis for the reasons mentioned above.

## Discussion

Findings in this study show that resting state connectivity between left and right M1, and WM integrity in the transcallosal M1–M1 tract are significant correlated with the UE-FM score. However, there is no significant relationship between resting state interhemispheric M1 connectivity and WM integrity in the transcallosal M1–M1 tract. Thus, functional and structural connectivity between interhemispheric motor cortex can be a marker of stroke motor impairment, but that they each have some independence from one another.

These findings support those that showed a similar relationship between motor ability and interhemispheric motor connectivity in patients <4 weeks post-stroke who were of a higher functioning level (5). Related, others have found that the early acute stage of stroke is marked by diminished resting state connectivity between left and right M1, this connectivity only increasing at 3–6 months post-stroke (6), presumably as patients gradually recover. One might speculate that this temporal coupling in neural activity may reflect the state of interhemispheric M1 inhibition. A commonly thought hypothesis is that ipsilesional M1 can no longer exert its normal inhibitory influence on contralesional M1 (18, 19) and/or the contralesional M1 exerts too much of an unbalanced inhibitory influence on ipsilesional M1 (20). Our findings suggest that this may be a possibility as patients with better motor recovery had higher interhemispheric M1 connectivity and thus, one might speculate, a more normal inhibitory influence of ipsilesional to contralesional M1. Future studies should aim to relate resting state connectivity with functional measures as assessed by TMS, tDCS, and/or fMRI to assess this possibility.

Fractional anisotropy is a sensitive but non-specific biomarker of microstructural architecture. To understand this measure in more detail, one can assess the components that comprise it, radial and axial diffusivity. Radial diffusivity is suggested to reflect the state of myelination of an axon while axial diffusivity may be a marker for axonal damage (21). Together, these measures have been used to characterize WM damage from stroke, among other disease pathologies. In our group of chronic stroke patients, WM integrity (i.e., FA) was reduced with an increase in radial diffusivity in the ipsilesional CST and the transcallosal M1–M1 tract compared to healthy controls. We also found that FA in the transcallosal M1–M1 tract was significant correlated with the UE-FM score: patients with lower FA and higher radial diffusivity values were more impaired. These findings are consistent with those from prior studies in chronic (3, 4) and acute (22, 23) stroke, and suggest that the FA decreases may be related to reduced integrity of the myelin sheath (21). For example, dead axons may lead to myelin disintegration that may consequently increase the space between the surviving fiber bundles, leading to increased radial diffusivity. In contrast, there were no detected differences in axial diffusivity in patients compared to controls. This may suggest that for remaining axons, remodeling may have sufficiently taken place and thus there are no detectable barriers to axial diffusion.

Despite the fact that interhemispheric M1 resting state connectivity and WM integrity correlate with the UE-FM score, there was no significant relationship between measures of functional and structural connectivity. In healthy subjects, it is thought that there is a close relationship between functional and structural connectivity measures (24). In particular, when structural connectivity is present, its integrity is related to the strength of functional connectivity (25). However, the presence of functional connectivity does not always reflect the presence of structural connectivity (26). To our knowledge, it is not known how functional and structural connectivity measures relate in stroke patients. For example, one patient in our study had a relatively high FA value despite being the subject with the lowest UE-FM score, and a relatively low resting state connectivity value. Thus it may be that having relatively intact WM integrity does not translate to the presence of meaningful functional signals passing through these axons and vice versa, having a few axons intact might be sufficient for functional signals to pass through. Related, a study of patients with amyotrophic lateral sclerosis also found an ambiguous relationship between functional and structural connectivity measures; functional connectivity was increased within regions that had reduced structural connectivity (27). Thus, future studies will need to investigate under what circumstances might a relationship between these two measures of connectivity exist or be absent, in clinical populations.

Lastly, we did not find any significant relationship between WM integrity in the ipsilesional CST and motor performance. This is in contrast to prior findings that showed significant correlations between FA in ipsilesional and contralesional CST with motor skill (3) and FA in the PLIC with motor skill during the acute (28, 29) and chronic (4, 30, 31) stage of stroke. However, this difference could be attributed to the fact that these prior studies examined patients that were higher functioning (i.e., patients performed the Purdue Pegboard test, ARAT, rapid index finger tapping, which are assessments of manual dexterity), and/or examined only a portion of the CST (i.e., the PLIC), and included lesioned voxels in their analyses. In contrast, we examined the entire non-lesioned CST tract in patients with a range of ability, and also only looked at voxels that were part of the “skeleton,” that is voxels that represent the center of the CST tract.

## Conclusion

Our findings show that interhemispheric M1 resting state connectivity and WM integrity is significantly correlated with UE-FM impairment. The importance of these functional and structural connections may relate to the degree in which contra- and ipsilesional motor regions interact with one another to facilitate motor stroke recovery. These findings enable us to target therapies (i.e., non-invasive brain stimulation) that may modulate existing neuronal connections or facilitate new connections and thus enhance recovery.

## Authors Contribution

Joyce L. Chen designed the study, collected the data, analyzed the data, and wrote the manuscript. Gottfried Schlaug provided assistance in designing the study, provided feedback on data analysis, and provided feedback on the manuscript.

## Conflict of Interest Statement

The authors declare that the research was conducted in the absence of any commercial or financial relationships that could be construed as a potential conflict of interest.

## Supplementary Material

The Supplementary Material for this article can be found online at http://www.frontiersin.org/journal/10.3389/fneur.2013.00178/abstract

Table S1 **Regions where controls have greater FA than patients**.

Table S2 **Regions where patients have greater axial diffusivity (L1) than controls**.

Table S3 **Regions where patients have greater radial diffusivity (L2/L3) than controls**.

Figure S1 **Top row shows white matter regions where controls have greater fractional anisotropy (FA) than patients**. Cluster numbers are indicated next to region and correspond to those listed in Table S1 in Supplementary Material. Second and third rows show white matter regions where patients have greater axial diffusivity (L1) than controls. Cluster numbers are indicated next to region and correspond to those listed in Table S2 in Supplementary Material. Rows four to six show white matter regions where patients have greater radial diffusivity (L2/L3) than controls. Cluster numbers are indicated next to region and correspond to those listed in S3. Images are taken in axial (*z*) or coronal (*y*) planes. All images show results that are significant at *t* > 3.1, *p* < 0.05 FWE using cluster thresholding.

## Footnotes

^1^http://www.fmrib.ox.ac.uk/fsl

^2^http://sites.google.com/site/phlemtoolbox/
