# Supplementary material for: Resting State Interhemispheric Motor Connectivity and White Matter Integrity Correlate with Motor Impairment in Chronic Stroke
Source: Front Neurol. 2013 Nov 7;4:178. doi: 10.3389/fneur.2013.00178 (PMC3819700; doi:10.3389/fneur.2013.00178)
Supplement: Table S1 — Regions where controls have greater FA than patients. [file 64716_Schlaug_Presentation1.PDF]

Supplemental Tables

S1. Regions where controls have greater FA than patients

Cluster	# Voxels	MNI coordinates (1mm space)			Regions encompassing cluster
		x	y	z	
1	920	-13	-2	56	WM under superior frontal gyrus, precentral gyrus, body of corpus callosum
2	897	-3	19	17	Body and genu of corpus callosum
3	871	-25	-24	8	Posterior limb of the internal capsule, cerebral peduncle
4	406	24	-39	30	Posterior corona radiata, body and splenium of corpus callosum
5	254	19	15	30	Superior and anterior corona radiata, genu of corpus callosum
6	176	-41	-47	9	Superior longitudinal fasciculus (posterior part)
7	144	-30	-41	25	Posterior corona radiata
8	138	-24	-59	24	Posterior thalamic radiation
9	119	-20	-58	-34	Cerebellum (WM near lobule VI)

10	104	-20	-87	1	Forceps major
11	101	24	-53	22	Forceps major
12	98	23	-3	36	Superior corona radiata
13	88	28	-57	-37	Cerebellum (WM near lobule VI)

Regions of significantly higher fractional anisotropy (FA) in controls compared to patients. Results are reported using cluster thresholding where $t > 3.1$, $p < 0.05$ FWE corrected. The first region within the cluster is reported in MNI coordinates.

S2. Regions where patients have greater axial diffusivity (L1) than controls

Cluster	# Voxels	MNI Coordinates (1 mm space)			Regions encompassing cluster
		x	y	z	
1	719	-19	32	14	Anterior corona radiata
2	215	28	-39	40	Superior longitudinal fasiculus
3	186	11	30	8	Genu of corpus callosum
4	167	24	-30	36	Posterior corona radiata
5	147	-12	-22	0	WM near thalamus
6	99	-31	-27	-4	Inferior longitudinal fasiculus
7	86	-25	-39	29	Posterior corona radiata
8	82	29	-15	43	Superior longitudinal fasciculus, WM under precentral gyrus
9	78	26	-7	43	Superior longitudinal fasiculus, WM under precentral gyrus
10	74	-16	12	41	WM under superior frontal gyrus
11	71	-25	-36	23	Posterior thalamic radiation
12	70	-21	-41	49	WM under sensorimotor cortex
13	69	40	-8	-22	Inferior longitudinal fasciculus
14	67	-17	34	24	Forceps minor
15	58	29	-23	40	Superior longitudinal fasiculus

Regions of significantly higher axial diffusivity in patients compared to controls. Results are reported using cluster thresholding where $t > 3.1$, $p < 0.05$ FWE corrected. The first region within the cluster is reported in MNI coordinates.

S3. Regions where patients have greater radial diffusivity (L2/L3) than controls

Cluster	# Voxels	MNI Coordinates (1 mm space)			Regions encompassing cluster
		x	y	z	
1	4110	-15	-15	55	WM under superior frontal gyrus, including forceps minor, genu of corpus callosum
2	1015	21	-33	49	WM under postcentral and precentral gyrus, superior corona radiata
3	901	-23	-20	14	Posterior limb of internal capsule, cerebral peduncle
4	570	27	-26	31	Posterior corona radiata
5	231	-36	-55	17	Posterior thalamic radiation
6	185	-22	-36	32	Splenium and body of corpus callosum
7	167	29	11	27	Superior corona radiata
8	139	-41	-57	5	Superior longitudinal fasciculus
9	138	-34	-66	3	Inferior longitudinal fasiculus
10	129	-40	-38	-12	Inferior longitudinal fasiculus
11	120	-111	-59	25	Splenium of corpus callosum, cingulum
12	117	29	-5	23	Superior corona radiata, superior longitudinal fasiculus
13	115	-31	41	6	Inferior fronto-occipital fasiculus
14	106	-20	-88	2	Forceps major
15	105	19	49	-2	Forceps minor and thalamic radiation

16	93	-28	-29	5	Fornix
17	83	4	-23	24	Body of corpus callosum
18	78	22	-87	4	Forceps major
19	71	19	35	-7	Inferior fronto-occipital fasiculus
20	68	-9	2	31	Cingulum
21	68	-31	-52	-38	Cerebellum, near lobule VI
22	57	30	-65	17	Forceps major

Regions of significantly higher radial diffusivity in patients compared to controls. Results are reported using cluster thresholding where $t> 3.1$, $p< 0.05$ FWE corrected. The first region within the cluster is reported in MNI coordinates.

Supplemental Figure

S1

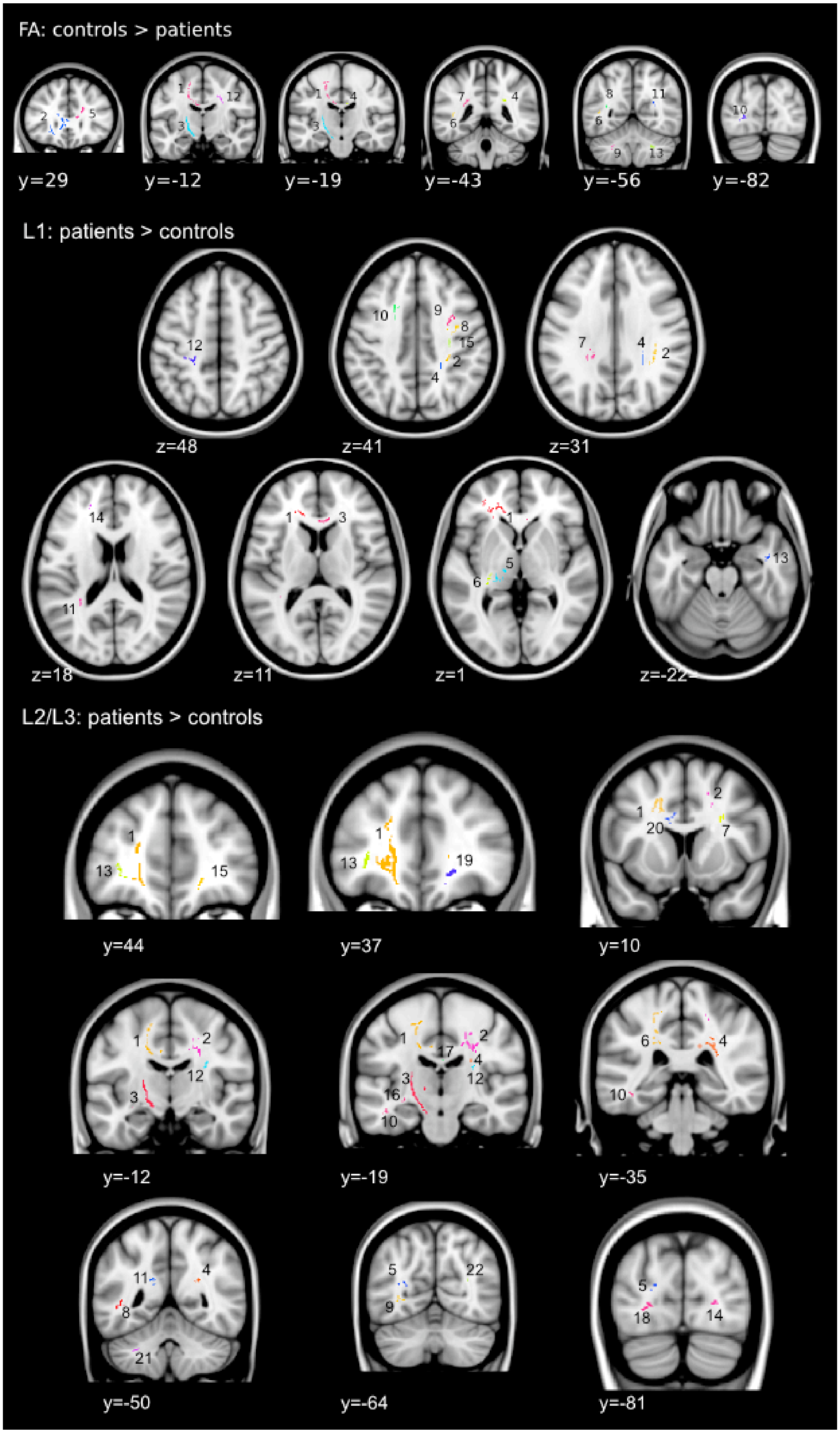
FA: controls > patients
y=29
y=-12
y=-19
y=-43
y=-56
y=-82
L1: patients > controls
z=48
z=41
z=31
z=18
z=11
z=1
z=-22
L2/L3: patients > controls
y=44
y=37
y=10
y=-12
y=-19
y=-35
y=-50
y=-64
y=-81

Top row shows white matter regions where controls have greater fractional anisotropy (FA) than patients. Cluster numbers are indicated next to region and correspond to those listed in S1. Second and third rows show white matter regions where patients have greater axial diffusivity (L1) than controls. Cluster numbers are indicated next to region and correspond to those listed in S2. Rows four to six show white matter regions where patients have greater radial diffusivity (L2/L3) than controls. Cluster numbers are indicated next to region and correspond to those listed in S3. Images are taken in axial (z) or coronal (y) planes. All images show results that are significant at $t>3.1$, $p<0.05$ FWE using cluster thresholding.
